# End-digits preference for self-reported height depends on language

**DOI:** 10.1186/1471-2458-8-342

**Published:** 2008-09-30

**Authors:** Matthias Bopp, David Faeh

**Affiliations:** 1 Institute of Social and Preventive Medicine (ISPM), University of Zurich, Hirschengraben 84, 8001 Zurich, Switzerland

## Abstract

**Background:**

When individuals report figures, they often prefer to round to specific end-digits (e.g. zero). Such preference has been found in reports of body weight, cigarette consumption or blood pressure measurements. Very little is known about self-reported body height. End-digit preference can distort estimates of prevalence and other statistical parameters. This study examines end-digit preference for self-reported height and how it relates with sex, age, educational level or cultural affiliation.

**Methods:**

We analysed reports of height of 47,192 individuals (aged 15 years or older) living in Switzerland and participating in one of the three population-based Swiss Health Surveys carried out in 1992/93, 1997 and 2002 respectively. Digit preferences were analysed by sex, age group, educational level, survey, smoking status, interview language (only for Swiss nationals) and nationality. Adjusted odds ratios (OR) with 95% confidence interval were calculated by using multivariate logistic regression.

**Results:**

Italian and French nationals (44.1% and 40.6%) and Italian and French Swiss (39.6% and 35.3%) more strongly preferred zero and five than Germans and German Swiss (29.2% and 30.3%). Two, four, six and eight were more popular in Germans and German Swiss (both 44.4%). Compared to German Swiss (OR = 1), for the end-digits zero and five, the OR were 1.50 (1.38–1.63) for Italian Swiss and 1.24 (1.18–1.30) for French Swiss; 1.73 (1.58–1.89) for Italian nationals and 1.61 (1.33–1.95) for French nationals. The end-digits two, four, six and eight showed an opposite pattern.

**Conclusion:**

Different preferences for end-digits depending on language and nationality could be observed consistently in all three national health surveys. The patterns were strikingly similar in Swiss and foreign nationals speaking the same language, suggesting that preferences were specific to language rather than to nationality. Taking into account rounding preferences could allow more valid comparisons in analyses of self-reported data originating from different cultures.

## Background

Body stature has an influence on various aspects of life including income, health related quality of life and on success in career and in mate selection [1-4]. Size is also inversely related with unintentional injury and mortality from cardiovascular disease and cancer [5-7]. Size can even decide on life and death on the battlefield [8]. Mostly, persons tend to overestimate their height when they report it [9,10]. Misreporting height varies by sex, age, education and by height status [10,11]. Differences between self-reported and measured height may result in differential misclassification of subjects thus leading to misinterpretation of the relationship between height and related outcomes [12]. Even a small measurement deviation in height can lead to large differences in the prevalence of derived categories [13,14].

Besides general overestimation, humans tend to round figures, e.g. to zero or five. This could also be the case for body height, but evidence is scarce [15-17]. Some information is available for end-digit preferences in reporting body weight, unfortunately without addressing end-digit preferences in height [18-20]. To our knowledge, it has never been described whether rounding preferences for height differ between men and women or depend on age, education or culture. This is an important issue, since end-digit preference can induce bias, when height-related prevalences within and between populations are compared, especially when thresholds matter. Studies have shown that rounding body weight to zero or five is associated with increased reporting error [19,21]. Thus, better knowledge on rounding preferences could be used to create indicators for data quality and help to develop correction factors that could be implicated to adjust reports of height.

Switzerland is partitioned in three well-defined linguistic areas and a large proportion of foreign nationals and is thus an ideal setting for the exploration of cultural influences. Confounding which commonly arises in international comparisons can be avoided, because the underlying data has been collected in an equal manner in all regions and subpopulations. Our aim was to investigate differences in preferences for end-digits in self-reported height and whether these preferences were related to specific subject characteristics. In particular we focus on patterns associated with language and nationality.

## Methods

We analysed data of persons aged 15 years and older who participated in one of three representative Swiss national health surveys (SHS, 1992/93, 1997, 2002 [22]) and reported body height in full centimetres (i.e. without decimals). The participation rates in the three SHS were 71%, 60% and 64% respectively. Data was gathered with the same method (by telephone interview) in all three surveys. All three surveys were sampled following a two-staged procedure (canton and household). Cantons were offered to increase their sample size at their own cost, an opportunity which was used in a variable extent. The participants were thereafter weighted according to the general population. For our analyses we used unweighted data and excluded 806 individuals with missing height or weight data. The final data set consisted of 47,192 persons. Characteristics of the three population samples are given in table 1.

**Table 1:** Study population: counts and (unweighted) proportions, Swiss Health Surveys (SHS) 1992–2002, (N = 47,192)

	SHS 1992/93		SHS 1997		SHS 2002	
			
	*N*	%	*N*	%	*N*	%
Total	14,899	100.0	12,821	100.0	19,472	100.0
						
Females	8,150	54.7	7,105	55.4	10,629	54.6
Males	6,749	45.3	5,716	44.6	8,843	45.4
						
Ages 15–30	3,556	23.9	2,650	20.7	2,810	14.4
Ages 31–45	4,554	30.6	4,097	32.0	6,049	31.1
Ages 46–60	3,367	22.6	2,772	21.6	4,836	24.8
Ages 60+	3,422	23.0	3,302	25.8	5,777	29.7
						
No secondary or tertiary education	3,445	23.1	3,065	23.9	4,148	21.3
Secondary education	8,312	55.8	7,606	59.3	12,132	62.3
Tertiary education	3,142	21.1	2,150	16.8	3,192	16.4
						
Swiss nationals, German-speaking	8,956	60.1	7,221	56.3	11,809	60.6
Swiss nationals, French-speaking	3,245	21.8	2,809	21.9	4,098	21.0
Swiss nationals, Italian-speaking	660	4.4	773	6.0	1,199	6.2
German^1 ^nationals	332	2.2	254	2.0	379	1.9
French nationals	175	1.2	124	1.0	152	0.8
Italian nationals	674	4.5	705	5.5	728	3.7
Other nationals	857	5.8	935	7.3	1,107	5.7
						
BMI <18.5	784	5.3	571	4.5	764	3.9
BMI 18.5–24.99	9,667	64.9	7,907	61.7	11,331	58.2
BMI 25–29.99	3,644	24.5	3,467	27.0	5,779	29.7
BMI 30+	804	5.4	876	6.8	1,598	8.2
						
Never-smokers	7,078	47.5	6,045	47.4	9,471	48.6
Ex-smokers	3,097	20.8	2,439	19.1	4,084	21.0
Smokers	4,720	31.7	4,270	33.5	5,913	30.4

^1 ^including nationals of Austria and the Principality of Liechtenstein

Participants were classified into four age groups (in years: 15–30, 31–45, 46–59, 60+), three educational levels (no secondary education, secondary education, tertiary education) and seven categories related to nationality and language: German, French or Italian-speaking Swiss nationals, nationals from other German-speaking countries (Germany, Austria, Principality of Liechtenstein), from France or Italy, and other foreign nationals). The categories of Swiss nationals were determined by the language in which the interview was conducted (irrespective of the place of residence, participants could choose whether they wanted to be interviewed in German, French or Italian). Body Mass Index (BMI=weight/(height)^2^) was calculated using self -reported height and weight and was divided in <18.5 kg/m^2 ^(underweight), 18.5–24.99 kg/m^2 ^(normal weight), 25.0–29.99 kg/m^2 ^(overweight), ≥ 30.0 kg/m^2 ^(obesity). The questions "Do you smoke" (yes), "What do you smoke" (cigarettes) and "How many cigarettes do you smoke" were combined to "current smokers" (≥ 1 cigarette/day). When the answer to the question "Do you smoke" was "no" and the answer to the question "have you ever smoked cigarettes longer than six months" was "yes", the persons were defined as "ex-smokers". The rest was defined as "never-smokers". For 75 participants smoking status was not available and they were excluded from multivariate regression. In separate analysis (results not shown) we tested whether the reclassification of persons with missing smoking status into one of the three smoking categories changed the figures presented here, but this was not the case.

In univariate analysis we used the χ^2 ^test to assess statistical significance. Odds ratios were calculated by using multivariate logistic regression with end-digit (e.g. digit zero: yes or no) or combinations of end-digits as dependent variable and adjusting for all listed variables. In order to get an idea about the "real" height distribution in the Swiss population, we analysed measured height data from the three MONICA studies (n = 10,144) which were carried out in Switzerland between 1984 and 1993 [23]. Analyses were performed with Stata 9.2 (Stata Corp, Texas, USA).

## Results

Table 1 shows the study population included in our analysis. As mentioned above the sampling fractions varied due to optional increase of sample size, but generally, this had little influence on the proportions. Participants were somewhat older in the SHS 1997 and (even more so) in the SHS 2002 than in the SHS 1992/93. In contrast, the percentage of persons with tertiary education was higher in SHS 1992/93 compared to the two other surveys. There were slightly fewer Italian-speaking persons in SHS 1992/93 than in the other surveys. Overall, there were no substantial differences between surveys in the composition of language and nationality. The proportion of overweight/obese persons increased over the three health surveys but the proportion of smokers remained relatively constant.

**Figure 1:**
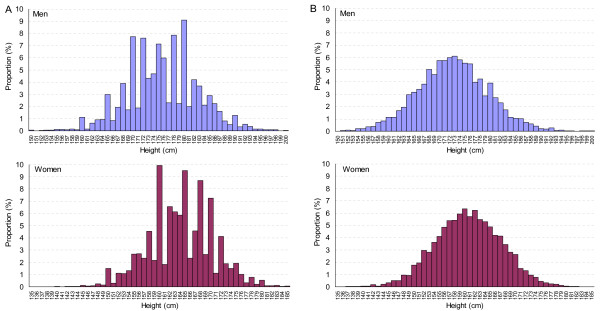
**Distribution of body height by sex. A: Swiss Health Surveys 1992–2002, (self-reported, N = 47,192), B: MONICA study, Switzerland 1984–1993, (measured, N = 10,144)**. MONICA: Monitoring Trends and Determinants in Cardiovascular Disease.

Figure 1A shows the distribution of self-reported heights in men and women which are used for our analysis. For a comparison with a distribution of measured height we used a dataset from the MONICA study (figure 1B). As shown in this figure, there is only negligible preference for end-digits in measured heights: in women 150 cm, 160 cm and 170 cm were marginally more frequent than may be expected, in men 164 cm, 168 cm, 170 cm, 176 cm and 180 cm. Compared to this "real" distribution, the distribution of self-reported heights shown in figure 1A is much less regular. Both men and women over-reported multiples of 10 (150 cm, 160 cm, 170 cm, 180 cm, 190 cm) and 5 (155 cm, 165 cm, 175 cm, 185 cm). Heights ending with two (162 cm, 172 cm, 182 cm) and with eight (158 cm, 168 cm, 178 cm) were also more frequently reported than expected. The preference for these figures appears independent of body height. Shorter men and women did not use the end-digits zero, five, two and eight substantially more often than taller men and women. Also, reported heights adjacent to the preferred figures were used similarly infrequent in shorter and taller individuals.

**Figure 2:**
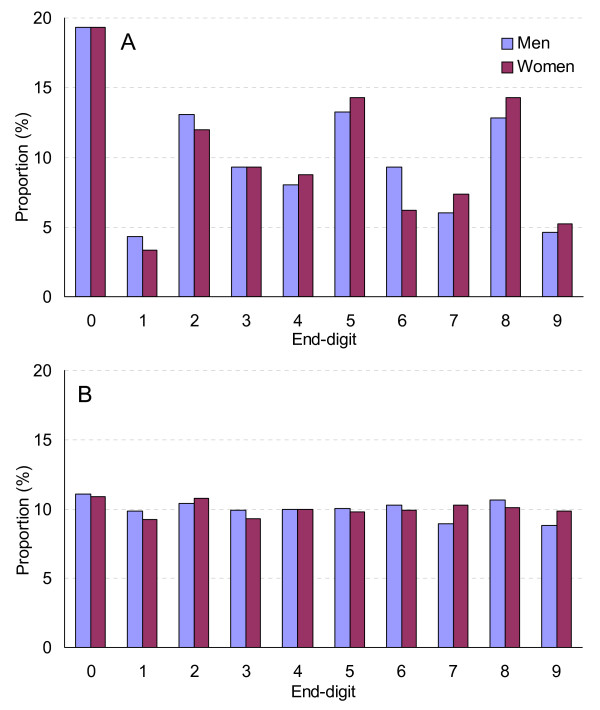
**Distribution of end-digits of height (cm) by sex. A: Swiss Health Surveys 1992–2002, (self-reported, N = 47,192), B: MONICA study, Switzerland 1984–1993, (measured, N = 10,144)**. MONICA: Monitoring Trends and Determinants in Cardiovascular Disease.

If measurement and reporting error was randomly distributed one could expect, that around 10% of the sample would report heights ending in each of the digits zero through nine. In fact, when height is measured each end-digit has a frequency close to 10% (figure 2B). This means, that in our sample, of those reporting height ending with zero (19.3%), 9.3% may be assumed to have misreported height (figure 2A). Following this rationale, 3.8% erroneously reported five, 3.6% eight and 2.5% two. Overall, it may be assumed that about 20% of all subjects rounded their height to the end-digits zero, two, five or eight (figure 2A). In men compared to women, the end-digits one (4.3% vs. 3.4%), two (13.1% vs. 12.0%) and six (9.3% vs. 6.2%) were chosen more frequently. In women compared to men, the end-digits four (8.7% vs. 8.0%), seven (7.4% vs. 6.0%), eight (14.3% vs. 12.8%) and nine (5.2% vs. 4.6%) were more popular. With respect to five and zero, preferences differed only slightly between men and women (32.6% vs. 33.6%).

Table 2 shows the distribution of end-digits (in absolute numbers and in %) by year of survey and overall. Zero and five were more frequently used by French and in particular by Italian-speaking Swiss nationals than by German-speaking Swiss, while the opposite was the case for the end-digits two, four, six and eight. Similar patterns were found in German vs. French and Italian Nationals. The figures were almost identical in German-speaking Swiss and Germans. Some difference was found between French-speaking Swiss and French nationals on the one hand and Italian-speaking Swiss and Italian nationals on the other hand for the end-digits zero and five and for the end-digits two, four, six and eight respectively, with somewhat stronger preference for the former and weaker preference for the latter in foreign than in Swiss nationals. However, the rank order of the language groups was identical in both Swiss and foreign nationals.

**Table 2:** Self-reported end-digits in height: Swiss Health Surveys (SHS) 1992–2002 (N = 47,192)

	Swiss Nationals: interview language											
				
End-digit preference	German			French			Italian					
						
	N	%		N	%		N	%				
			
SHS 1992/93	8,956	100.0		3,245	100.0		660	100.0				
0	1,715	19.1	**	676	20.8		156	23.6				
5	1,179	13.2	**	504	15.5	*	117	17.7	*			
8	1,253	14.0		408	12.6		80	12.1				
2	1,198	13.4		399	12.3		78	11.8				
0+5	2,894	32.3	**	1,180	36.4		273	41.4	**			
2+4+6+8	3,918	43.7	**	1,292	39.8		242	36.7	*			
SHS 1997	7,221	100.0		2,809	100.0		773	100.0				
0	1,231	17.0	**	554	19.7		195	25.2	**			
5	883	12.2	**	443	15.8	**	112	14.5				
8	1,058	14.7	**	339	12.1	*	96	12.4				
2	918	12.7		316	11.2		79	10.2				
0+5	2,114	29.3	**	997	35.5	*	307	39.7	**			
2+4+6+8	3,222	44.6	**	1,105	39.3	*	273	35.3	**			
SHS 2002	11,809	100.0		4,098	100.0		1,199	100.0				
0	2,047	17.3	**	787	19.2		288	24.0	**			
5	1,435	12.2	**	615	15.0	**	175	14.6				
8	1,708	14.5		564	13.8		149	12.4				
2	1,538	13.0		488	11.9		131	10.9				
0+5	3,482	29.5	**	1,402	34.2	*	463	38.6	**			
2+4+6+8	5,273	44.7	**	1,657	40.4	*	455	37.9	*			
Total (all 3 SHSs)	27,986	100.0		10,152	100.0		2,632	100.0				
0	4,993	17.8	**	2,017	19.9		639	24.3	**			
5	3,497	12.5	**	1,562	15.4	**	404	15.3	*			
8	4,019	14.4	**	1,311	12.9		325	12.3				
2	3,654	13.1	**	1,203	11.8		288	10.9	*			
0+5	8,490	30.3	**	3,579	35.3	**	1,043	39.6	**			
2+4+6+8	12,413	44.4	**	4,054	39.9	**	970	36.9	**			
	Foreign Nationals (irrespective of interview language)											
	
End-digit preference	Germany^1^			France			Italy			Other		
				
	N	%		N	%		N	%		N	%	

SHS 1992/93	332	100.0		175	100.0		674	100.0		857	100.0	
0	67	20.2		43	24.6		195	28.9	**	192	22.4	
5	39	11.7		25	14.3		125	18.5	**	131	15.3	
8	43	13.0		20	11.4		80	11.9		109	12.7	
2	40	12.0		18	10.3		62	9.2	**	113	13.2	
0+5	106	31.9		68	38.9		320	47.5	**	323	37.7	
2+4+6+8	140	42.2		66	37.7		230	34.1	**	342	39.9	
SHS 1997	254	100.0		124	100.0		705	100.0		935	100.0	
0	45	17.7		35	28.2	*	153	21.7		210	22.5	*
5	25	9.8		20	16.1		139	19.7	**	156	16.7	*
8	36	14.2		18	14.5		75	10.6	*	99	10.6	*
2	25	9.8		8	6.5		91	12.9		115	12.3	
0+5	70	27.6		55	44.4	*	292	41.4	**	366	39.1	**
2+4+6+8	111	43.7		46	37.1		265	37.6		361	38.6	
SHS 2002	379	100.0		152	100.0		728	100.0		1,107	100.0	
0	67	17.7		39	25.7	*	180	24.7	**	234	21.1	
5	39	10.3		21	13.8		137	18.8	**	174	15.7	*
8	63	16.6		12	7.9	*	82	11.3		137	12.4	
2	53	14.0		20	13.2		72	9.9	*	128	11.6	
0+5	106	28.0		60	39.5		317	43.5	**	408	36.9	**
2+4+6+8	177	46.7		50	32.9		243	33.4	**	410	37.0	**
Total (all 3 SHSs)	965	100.0		451	100.0		2,107	100.0		2,899	100.0	
0	179	18.5		117	25.9	**	528	25.1	**	636	21.9	**
5	103	10.7	**	66	14.6		401	19.0	**	461	15.9	**
8	142	14.7		50	11.1		237	11.2	**	345	11.9	*
2	118	12.2		46	10.2		225	10.7	*	356	12.3	
0+5	282	29.2	*	183	40.6	**	929	44.1	**	1,097	37.8	**
2+4+6+8	428	44.4		162	35.9	*	738	35.0	**	1,113	38.4	**

^1 ^including nationals of Austria and the Principality of Liechtenstein

Counts (N) and proportions (%) in Swiss and foreign residents. Asterisks indicate statistical significant difference (respective linguistic group vs. all others, *: p < 0.05; **: p < 0.01)

Results of fully adjusted logistic regression analysis are shown in table 3. No significant gender difference was found for the end-digits zero and five, while the even numbers two, four, six and eight were less frequently used by women compared to men. Age had a stronger influence on the preference for even numbers than on the preference for the end-digits zero and five while the opposite was the case for educational level. Persons with lower education preferred zero and five rather than even numbers. No significant difference in any end-digit preference was found with respect to the four BMI categories. Smokers and ex-smokers reported the end-digits zero and five less often and even numbers more often than never-smokers but the differences were small. Differences between the surveys were negligible: only for the end-digits zero and five, there was a significantly higher OR for the SHS 1992/93.

**Table 3:** Preference for end-digits in height: Swiss Health Surveys (SHS) 1992–2002, (N = 47,117).

End-digit preference	0 or 5				2, 4, 6 or 8			
		
	%	OR	LCI	UCI	%	OR	LCI	UCI
Men	32.5	1			43.2	1		
Women	33.5	1.00	0.96	1.04	41.2	0.94	0.90	0.97
								
Age 15–30 y	35.1	1.11	1.05	1.18	39.0	0.84	0.79	0.89
Age 31–45 y	32.5	1.06	1.00	1.12	41.3	0.90	0.86	0.95
Age 46–59 y	31.3	1			43.9	1		
Age 60+y	33.7	1.09	1.03	1.15	43.8	1.01	0.96	1.07
								
No secondary or tertiary education	37.9	1.29	1.21	1.38	39.7	0.91	0.86	0.97
Secondary education	32.0	1.08	1.02	1.14	42.7	0.99	0.94	1.04
Tertiary education	30.5	1			43.3	1		
								
Swiss nationals, German-speaking	30.3	1			44.4	1		
Swiss nationals, French-speaking	35.3	1.24	1.18	1.3	39.9	0.84	0.80	0.88
Swiss nationals, Italian-speaking	39.6	1.50	1.38	1.63	36.9	0.73	0.68	0.80
German^1 ^nationals	29.2	0.98	0.85	1.13	44.4	0.98	0.86	1.12
French nationals	40.6	1.61	1.33	1.95	35.9	0.70	0.58	0.85
Italian nationals	44.1	1.73	1.58	1.89	35.0	0.69	0.63	0.76
Other nationals	37.8	1.34	1.24	1.46	38.4	0.83	0.76	0.90
								
BMI <18.5	35.7	1.07	0.98	1.18	40.8	1.03	0.94	1.13
BMI 18.5–24	33.1	1			41.5	1		
BMI 25–29	32.8	0.99	0.94	1.03	43.4	1.03	0.99	1.08
BMI ≥ 30	31.9	0.94	0.87	1.02	43.5	1.05	0.97	1.13
								
Never-smokers	34.4	1			41.1	1		
Ex-smokers	31.4	0.90	0.86	0.94	43.5	1.09	1.04	1.13
Smokers	32.1	0.89	0.85	0.94	42.8	1.06	1.00	1.11
								
Survey 1992/93	34.7	1.12	1.07	1.17	41.8	0.99	0.95	1.04
Survey 1997	32.8	1.01	0.96	1.06	42.0	1.01	0.96	1.06
Survey 2002	32.0	1			42.4	1		

^1 ^including nationals of Austria and the Principality of Liechtenstein

Proportions (%) and odds ratios (OR, adjusted for all other listed variables), with upper (UCI) and lower (LCI) 95% confidence interval.

## Discussion

Analysing self-reported height in 47,192 residents of Switzerland, we found significant variations in preferences for specific end-digits. These preferences were more specific to language than to nationality. Overall, zero and five have been reported more frequently (33.1%) than could be expected by chance alone (i.e. 20%). Similar preferences for end-digits zero and five when reporting height were found by others [15,16]. Interestingly, rounding height also appears to depend on measurement system. In a study carried out in Australia, 41.2% of the reports in metric system had an end-digit zero or five, while only 18.4% of those reporting their height in feet and inches had zero or six inches as end-digit [16]. Except of height, end-digit preference was reported for blood pressure (zero, five and eight) [24], daily cigarette consumption (multiples of 10 cigarettes) [25] and weight (zero and five) [18,19,21,26,27].

In our study, the middle-aged were those least reporting the end-digits zero and five. Others found that reporting these end-digits for body weight increased with age [21]. In analogy to our findings in height, others showed higher proportions of persons with low educational level using end-digits zero and five for reporting smoking [25] or for body weight [18,21]. However, other studies found no association between end-digit preference for weight and education [19,26,27]. Shorter subjects may be more prone to over-report height [15,19] but we cannot determine (not based on our dataset [Figure 1A], nor from literature), whether rounding of end-digits goes along with higher under/overestimation of height. In measured height of Swiss conscripts (complete coverage), a larger difference in mean height between German and Italian speaking men than in the SHS data can be observed [28]. However, no similar pattern can be discerned when comparing German and French speaking men.

Interestingly, we found that preferences for end-digits were strikingly similar in Swiss and foreign nationals speaking the same language (e.g. French-speaking Swiss and French nationals), suggesting that this preference is specific to language rather than to nationality. This is not mainly due to a regional effect, since the vast majority of Italians in Switzerland do not live in the Italian-speaking part of the country. Intriguingly, these effects of language and nationality could be observed consistently in all three SHS, suggesting a strong and stable effect.

We are not aware of any study exploring cultural preferences in reporting end-digits. With respect to body weight, there were reports about differences in preferences for zero and five between ethnicities (preference was greater among women who were non-White compared to White) [21,27]. This was however not confirmed by others [19,26]. In a study analyzing digit preferences in reporting daily cigarettes smoked, Caucasians were more likely to round to multiples of ten than were Afro-Americans [25]. Mexican Americans were reported to underestimate overweight more substantially compared to African Americans and non-Hispanic European Americans, but unfortunately the study does not mention, whether this bias was generated by selective end-digit preference [29]. More information on potentially language-related influences in self-reports of height could be useful for evaluating anthropometric trends in culturally mixed populations, all the more when there are subpopulations which have rapidly increased in the past years (e.g. Latinos in the U.S.).

Digit preference could also be used as an indicator of quality control [30]. Non-directional reporting error in self-reported height has been associated with lower education, adolescent and old age and being a smoker [9,18]. Such reporting error would correspond in our study to persons with higher ORs for end-digits zero or five. Thus, if these end-digits were interpreted as a marker for poor reliability, one could suggest that reporting in French and even more so in Italian-speaking individuals was less reliable than in German-speakers. In contrast, directional reporting error (i.e. reporting a taller height than the real height) may increase with increasing age and BMI and may be higher in men [10]. Accordingly to these findings, it could be suggested, that in our analysis, this pattern of over-reporting height is associated with more frequent report of even end-digits (i.e. two, four, six or eight). We can however not determine whether German-speaking populations were more prone to over-report height than the other language groups. In our study, both German-speaking populations had distinctly lower prevalences of the end-digits zero and five than was reported in France [15] and Australia [16]. Frequent use of the end-digits zero and five could be interpreted as impreciseness or as low conscientiousness. A study assessing self-reported stereotypes about personality characteristics found highest self-rated conscientiousness in German Swiss, followed by Germans, French Swiss, French and Italians [31]. These results perfectly mirror our findings.

However all these assumption remain rather speculative, since we did not have the measured pendant to the self-reported heights. Nevertheless, others reported that men and women who rounded their height tended to overestimate it [15] and that reliability may decrease with decreasing real height [18]. Given the distinctly different report patterns in the major cultural groups of Switzerland, it appears probable that underlying cultural ideals and norms exist. Future studies should focus on the nature of the self-report bias [25].

Admittedly, we cannot prove that the "real" proportion of each end-digit (zero through nine) is 10 %, although this is biologically plausible. Many studies with measured heights showed proportions of end-digits close to 10% [17]. In this respect, the MONICA data of measured height, which we used for comparison, can be interpreted as rather precise. Another limitation concerns selection bias which is inherent in all surveys. Although the participation rate was fairly high in all three health surveys, we expect that participants represent a selection of health-conscious and rather well educated individuals. However, since in our and other [9,18] analyses lower education was associated with more frequent preference for specific end-digits, we assume that end-digit preference would have been even more pronounced had the participation rate been 100%.

## Conclusion

In Switzerland, preferences for end-digits in self-reported height significantly varied by age, sex and education. Preferences also differed between German-speaking persons on the one hand and Italian and French-speaking persons on the other hand, irrespective of nationality. These finding implicates that it may be difficult to obtain similarly valid self-reports of height in specific segments of the adult population (e.g. persons who speak a Romance language, women and those with little formal education). Our findings may also help to explain why reports on differential misreporting of height – often originating from different cultural settings – lack consistency [10]. Future studies should evaluate other potential confounders (e.g. social desirability, degree of health consciousness).

Better knowledge of cultural preferences in reporting numbers could help avoiding misclassification of individuals and should be taken into account when comparing self-reported data from different cultures. Periodical assessments of digit preference may also offer the potential for controlling and optimizing measurement quality and for creating adjustment factors for self-reported figures. This may be particularly important, when rounding to preferred end-digits impacts on treatment, e.g. in the case of blood pressure measurement and management of hypertension [32].

## Authors' contributions

MB conceived the study and sketched a first draft. DF prepared the data files of the three surveys used and wrote up the final manuscript. Search for literature, data analysis and interpretation of the results have been made by both authors, as well as repeated readings and improvements of the manuscript.

## Pre-publication history

The pre-publication history for this paper can be accessed here:


